# Supplementary material for: Pharmacokinetics of Moxidectin combined with Albendazole or Albendazole plus Diethylcarbamazine for Bancroftian Filariasis
Source: PLoS Negl Trop Dis. 2023 Aug 24;17(8):e0011567. doi: 10.1371/journal.pntd.0011567 (PMC10482275; doi:10.1371/journal.pntd.0011567)
Supplement: S1 Table — (DOCX) [file pntd.0011567.s001.docx]

**Pharmacokinetics of Moxidectin Combined with Albendazole or Albendazole plus Diethylcarbamazine for Bancroftian Filariasis**

Yashpal S Chhonker^1^, Catherine Bjerum^2^, Veenu Bala^1^, Allassane F. Ouattara^3^, Benjamin G. Koudou^3^_,_ Toki P Gabo^4^, Abdullah Alshehri^1^, Abdoulaye Meïté^5^, Peter U. Fischer^6^, Gary J. Weil^6^, Christopher L. King^2,7^ Philip J Budge^6^, and Daryl J. Murry^1, 8^

^1^Clinical Pharmacology Laboratory, Dept of Pharmacy Practice and Science, University of Nebraska Medical Center, Omaha, Nebraska, United States of America

^2^ Center for Global Health and Diseases, Case Western Reserve University School of Medicine, Cleveland, Ohio, United States of America

^3^ Centre Suisse de Recherche Scientifique en Côte d’Ivoire (CSRS), Abidjan, Ivory Coast

^4^Centre Hospitalier Regional d’Agboville, Côte d’Ivoire

^5^ Programme National de la Lutte Contre la Schistosomiase, les Geohelminthiases et la Filariose Lymphatique, Ivory Coast

^6^ Infectious Diseases Division, Department of Medicine, Washington University School of Medicine, St. Louis, Missouri, United States of America

^7^ Veterans Affairs Research Service, Cleveland Veterans Affairs Medical Center, Cleveland, Ohio, United States of America

^8^Fred and Pamela Buffett Cancer Center, University of Nebraska Medical Center, Omaha, Nebraska, United States of America

**Keywords:** Bancroftian Filariasis, lymphatic filariasis, mass drug administration, moxidectin

**Clinical Trials Registration.** NCT04410406

**Corresponding author:**

*[dj.murry@unmc.edu](mailto:dj.murry@unmc.edu)

**Supplementary Data**

**S1 Table : Pharmacokinetic parameters of the study drugs when administered in IDA, IA, MoxDA and MoxA study Arms.**

| **Drugs** | **ARMS** | **C_max_ (ng/mL)** | **T_max_ (hr)** | **Half-life (t_1/2_)** | **AUC_0-t_ (ng*hr/mL)** | **AUC_0–∞_ (ng*hr/mL)** | **V_z/F_ (L)** | **Cl/F (L/hr)** |
| --- | --- | --- | --- | --- | --- | --- | --- | --- |
| ALB-SOX | IDA | 345.5 (81.9) | 6 (14.8) | 7.1 (27.7) | 3437.8 (80.5) | 3438.6 (80.3) | 1046.4 (65.9) | 116.3 (57) |
|  | IA | 306.8 (76.3) | 4 (20.8) | 7.8 (24.9) | 3957.6 (87.8) | 3963.2 (87.5) | 1181.6 (50) | 100.9 (45.6) |
|  | MoxDA | 324.4 (42.6) | 6 (18.8) | 8.6 (36.5) | 4180.4 (44.4) | 4232.2 (44.5) | 1139.8 (65.5) | 94.7 (48.4) |
|  | MoxA | 433.2 (70.7) | 4 (25.3) | 9.4 (21.7) | 5743.2 (70.6) | 5826 (70.3) | 1033.1 (79.1) | 72.1 (76.2) |
| DEC | IDA | 1213 (16.4) | 4 (24.4) | 10.4 (27.3) | 21217.2 (23.5) | 21340.8 (24.8) | 136.7 (26.1) | 8.4 (24.3) |
|  | MoxDA | 1117.1 (18.7) | 4 (55.1) | 10.7 (13.8) | 20681.7 (20.7) | 20843.3 (21) | 148.7 (19.6) | 9.7 (26.4) |
| IVM | IDA | 40.6 (48.5) | 6 (37.4) | 45.3 (41.4) | 1402.1 (41.3) | 1639.3 (49.6) | 393.8 (44.2) | 7.3 (31.9) |
|  | IA | 55.6 (59) | 6 (28.8) | 52.6 (64.4) | 1267.2 (50.4) | 1403.3 (69.8) | 554.8 (40) | 8.8 (53.8) |
| MOX | MoxDA | 108.3 (83.3) | 6 (24.8) | 56.8 (75.4) | 2168.1 (68) | 2619.4 (78.4) | 186.7 (84.4) | 3.1 (50.3) |
|  | MoxA | 94.2 (62.8) | 6 (17.3) | 93.1 (36.3) | 2134.1 (61.8) | 2631.5 (66) | 422.9 (67.4) | 3.1 (73.9) |

Data presented are the median (%CV) values for each pharmacokinetic parameter.

T_1/2_ terminal half-life, T_max_ time of maximum plasma concentration, C_max_ maximum plasma concentration, AUC area under the concentration-time curve, Vz/F apparent volume of distribution, CL/F apparent clearance.

ALB-OX, albendazole sulfoxide; ALB-ON, albendazole sulfone; DEC, diethylcarbamazine; IVM, ivermectin; MOX, Moxidectin.

IDA, three-drug combination (DEC 6mg/kg+ IVM 200ug/kg + ALB 400mg); IA, two-drug combination (IVM 200μg/kg + ALB 400mg); MOXDA, three drug combination (DEC 6mg/kg+ MOX 8mg + ALB 400mg); and MOXA, two-drug combination (MOX8 + ALB 400mg).
