# Supplementary material for: Pharmacokinetics of Moxidectin combined with Albendazole or Albendazole plus Diethylcarbamazine for Bancroftian Filariasis
Source: PLoS Negl Trop Dis. 2023 Aug 24;17(8):e0011567. doi: 10.1371/journal.pntd.0011567 (PMC10482275; doi:10.1371/journal.pntd.0011567)
Supplement: Attachment — Submitted filename: Reviewer comments response_ draft-6_20230723DJ.docx [file pntd.0011567.s002.docx]

**Responses to reviewer**

All comments by the reviewers have been addressed and updated in the manuscript via track changes text. Detailed responses to the reviewer’s comments are included below. We would like to take this opportunity to express our sincere gratitude to the reviewers who identified areas of our manuscript, which required improvement or further clarification. With these modifications, our manuscript has been strengthened in scientific merit and reads more professionally.

Reviewer #1: The objective of the study “…is to determine whether the incorporation of moxidectin (MOX) into MDA regimens for LF will alter the pharmacokinetics of co-administered drugs”. However, this is a sub study and this was not the primary objective of the clinical trial.

In terms of moxidectin’s risk of drug:drug interactions (DDIs), these were formally evaluated as part of its development by Wyeth/Pfizer, MDGH and the US FDA. Based on the metabolic pathways, transporters, and previous data with midazolam as a probe (Korth-Bradley et al, 2014), the risk of DDIs with this combination of compounds was negligible based on in silico modelling. While the paper describes data that are supportive of this position, this was not prospectively designed as a drug:drug interaction study (i.e clinical DDI studies compare substrate concentrations in the absence and presence of a perpetrator or victim drug). As such, negative findings (in terms of not identifying a DDI) from retrospective studies such as this (i.e not prospectively defined solely for the purpose of DDI evaluation) have limited value and cannot inform regulatory authorities. Recommend that the authors review FDA Guidance for Industry [https://urldefense.com/v3/__https://www.fda.gov/media/134581/download__;!!JkUDQA!JKxEINvHZyphJupArQO9tYMbSsO_EbC9_mlbvyifuKyCSOfJHvxW1G30TRqHv_boJyijug2S4Us5g_p4-XQ$](https://urldefense.com/v3/__https:/www.fda.gov/media/134581/download__;!!JkUDQA!JKxEINvHZyphJupArQO9tYMbSsO_EbC9_mlbvyifuKyCSOfJHvxW1G30TRqHv_boJyijug2S4Us5g_p4-XQ$) to help understand the deficiencies between this design and a DDI study.

**Reply:** We thank the reviewer for providing this informative document. We agree with the reviewer that DDI was not the primary objective of the study. Our results focus on potential alterations of the pharmacokinetics of drugs recommended for mass drug administration for lymphatic filariasis. As such, our results confirm the addition of moxidectin did not result in significant changes to the pharmacokinetics of these agents. We have clarified this in the discussion. This is critical information for the WHO to roll out these studies in a larger capacity and was the framework for the MDGH 3002 study evaluating IVM+ALB vs MOX+ALB in 4000 individuals. Without the PK data presented in this manuscript, the WHO would not have allowed the MDGH 3002 study to proceed**.**

A comment on how patients were selected for the pharmacokinetic sub study and how similar or different they were to the broader population in the clinical trial would also be helpful.

Reply: Patients who consented to the pharmacokinetic blood sampling were included in this sub study. The first 58 subjects consented to the substudy and demographics were consistent with the group, a total of 121 subjects were enrolled in the clinical protocol. A line has been added in results.

--------------------

**Reviewer #1**: The pharmacokinetic results of moxidectin and ivermectin are different from previously published data - ivermectin T1/2 is 15 hours in the US FDA PI and moxidectin’s is between 20 and 40 days. Ivermectin’s T1/2 was significantly longer than published data and moxidectin’s significantly shorter. Data from doi: 10.1371/journal.pntd.0010005 should be the primary comparison used in the discussion.

Reply: In our study, we collected blood samples out to 7 days post administration and the data analysis was conducted using non-compartmental methods. While the half life for ivermectin is 15 hours in the FDA PI, in studies of the drug in patients with Onchocerciasis receiving 150 ug/kg the half-life ranged from 16 hours to 54.5 hours. Previous studies in patients with LF by other groups have found the ivermectin half life be 46.5 hours (sd 38.7) (CID 2016:62 (1 February) • Thomsen et al) Our results in patients with LF are consistent with reported values.

For moxidectin, the previously reported pharmacokinetic study sampled out much longer than our study (out to 12 months) and used a non-specific method for analysis (fluorescence detection) while our method is a specific and validated LC-MS/MS method and our sample strategy only was out to 7 days. It is clear after approximately 200 hours, there is a prolonged half-life for moxidectin (or metabolite as fluorescence detection cannot distinguish between the two), however we did not sample out that long. We digitized the figures from the referenced manuscript that was presented (Digit 1.0.4 software) and determined the half life (using data to 8 days). Geometric mean concentrations at 8 days (in the referenced paper) ranged from 0.73 to 1.97 ng/mL while the assay limit of detection was 0.08 ng/mL. It is unclear the therapeutic value of moxidectin (or metabolite) concentrations less than 1 ng/mL even with and extremely long half-life. For the referenced paper and using only the values presented out to 8 days, the calculated half life values ranged from 45 hours (8 mg dose) to 66.3 hours (4 mg dose). The value for the 2 mg dose was 52 hours. It is clear the authors in that study used moxidectin values out to 1 year to calculate half-life. Without using a specific LCMS/MS method and only utilizing fluorescence detection, it is unclear if the late values represent metabolites or parent drug. Our half-life value using data to 7 days is consistent between studies and part of the difference is sampling time, but also specificity of analytical methods. Utilizing data to 7 days (our study) or 8 days (the suggested reference study and the only data presented) the half-life is consistent, and less than the reported 23 days.

Performance of the assays used should be commented upon - how the assays were validated should be commented on. Also, their performance (e.g. range of quantation, inter day, inter operator performance parameters should be given).

Reply: All validated bioanalytical methods have been previously reported from our laboratory and are referenced in this manuscript with no deviation on process. **The range of quantitation and performance parameters have been incorporated in the manuscript as suggested.**

The number of available samples at each time point should be noted at some place in the paper - were these analyses based on available samples or on patients with complete sets? Also, were the samples all compliant with sampling windows and if not, how many were deviant from prospectively defined windows?

Reply- All samples were compliant with sampling windows within a few minutes. All subjects had complete (ie 12) blood samples out to 7 days. **(IDA, n = 15, IA, n = 15, MOXDA, n = 14, MOXA, n = 14). The number of subjects in each arm has been included as footnote of figure 1.**

How close was dosing relative to food intake?

Reply: Timing and type of breakfast is listed page 6, the drugs were administered approximately 30 minutes following the breakfast meal and serial samples (listed on page 6) and serial samples were collected out to 7 days.

--------------------

Reviewer #1: The conclusions are supported but there is a tendancy to overstate the value of the data. This study was not the designed by standard DDI methodologies to evaluate the question. The outlying data from this study compared to all other pharmacokinetic data on moxidectin and ivermectin warrant comment - it leads to the differences being due to disease questions or study execution/assay questions. The underlying message remains that the data do not generate concerns about the use of these agents together.

**Reply:** Thank you for the comment – we have clarified this on page 16 and 17 of the discussion.

--------------------

Reviewer #1:

It should be noted that DEC can result in life threatening system reactions and not just ocular reactions in onchocerciasis patients.

Reply: This has been added to the text on page 4.

It should say that this is a sub study.

**Reply:** We included this information in the text (page 9).

--------------------

**Reviewer #1**: The data are of value. The comments on the experiment are already noted but the authors are to be congratulated on executing this trial.

**Reply:** We thank the reviewer for their positive comments and suggestions.

--------------------

PLOS authors have the option to publish the peer review history of their article (<a href="[https://urldefense.com/v3/__https://journals.plos.org/plosntds/s/editorial-and-peer-review-process*loc-peer-review-history__;Iw!!JkUDQA!JKxEINvHZyphJupArQO9tYMbSsO_EbC9_mlbvyifuKyCSOfJHvxW1G30TRqHv_boJyijug2S4Us5ZndhE1M$](https://urldefense.com/v3/__https:/journals.plos.org/plosntds/s/editorial-and-peer-review-process*loc-peer-review-history__;Iw!!JkUDQA!JKxEINvHZyphJupArQO9tYMbSsO_EbC9_mlbvyifuKyCSOfJHvxW1G30TRqHv_boJyijug2S4Us5ZndhE1M$)" target="_blank">what does this mean?</a>). If published, this will include your full peer review and any attached files.<br><br>

If you choose “no”, your identity will remain anonymous but your review may still be made public.<br><br>

<b>Do you want your identity to be public for this peer review?</b> For information about this choice, including consent withdrawal, please see our <a href="[https://urldefense.com/v3/__https://www.plos.org/privacy-policy__;!!JkUDQA!JKxEINvHZyphJupArQO9tYMbSsO_EbC9_mlbvyifuKyCSOfJHvxW1G30TRqHv_boJyijug2S4Us5dbuvWcA$](https://urldefense.com/v3/__https:/www.plos.org/privacy-policy__;!!JkUDQA!JKxEINvHZyphJupArQO9tYMbSsO_EbC9_mlbvyifuKyCSOfJHvxW1G30TRqHv_boJyijug2S4Us5dbuvWcA$)" target="_blank">Privacy Policy</a>.

Reviewer #1: No
